# Metabolic reprogramming, autophagy, and ferroptosis: Novel arsenals to overcome immunotherapy resistance in gastrointestinal cancer

**DOI:** 10.1002/cam4.6623

**Published:** 2023-10-20

**Authors:** Xiangwen Wang, Liwen Zhou, Hongpeng Wang, Wei Chen, Lei Jiang, Guangtao Ming, Jun Wang

**Affiliations:** ^1^ Department of General Surgery The First Hospital of Lanzhou University Lanzhou China; ^2^ Department of Stomatology The First Hospital of Lanzhou University Lanzhou China

**Keywords:** autophagy, ferroptosis, gastrointestinal cancer, immunotherapy resistance, metabolic reprogramming

## Abstract

**Background:**

Gastrointestinal cancer poses a serious health threat owing to its high morbidity and mortality. Although immune checkpoint blockade (ICB) therapies have achieved meaningful success in most solid tumors, the improvement in survival in gastrointestinal cancers is modest, owing to sparse immune response and widespread resistance. Metabolic reprogramming, autophagy, and ferroptosis are key regulators of tumor progression.

**Methods:**

A literature review was conducted to investigate the role of the metabolic reprogramming, autophagy, and ferroptosis in immunotherapy resistance of gastrointestinal cancer.

**Results:**

Metabolic reprogramming, autophagy, and ferroptosis play pivotal roles in regulating the survival, differentiation, and function of immune cells within the tumor microenvironment. These processes redefine the nutrient allocation blueprint between cancer cells and immune cells, facilitating tumor immune evasion, which critically impacts the therapeutic efficacy of immunotherapy for gastrointestinal cancers. Additionally, there exists profound crosstalk among metabolic reprogramming, autophagy, and ferroptosis. These interactions are paramount in anti‐tumor immunity, further promoting the formation of an immunosuppressive microenvironment and resistance to immunotherapy.

**Conclusions:**

Consequently, it is imperative to conduct comprehensive research on the roles of metabolic reprogramming, autophagy, and ferroptosis in the resistance of gastrointestinal tumor immunotherapy. This understanding will illuminate the clinical potential of targeting these pathways and their regulatory mechanisms to overcome immunotherapy resistance in gastrointestinal cancers.

## 1 INTRODUCTION

Gastrointestinal cancer is a major public health problem worldwide, with 26% and 35% of the total global cancer incidence and mortality, respectively.^1^ Although conventional treatments such as radiotherapy, chemotherapy, and surgery have been widely applied in treating gastrointestinal cancers, these strategies have modestly improved survival, especially in patients with advanced disease.^2^ In the past few years, the advent of immunotherapy has significantly changed the landscape of solid tumor treatment, and immune checkpoint blockade (ICB) therapies (e.g., anti‐PD‐1, anti‐PD‐L1, and anti‐CTLA‐4) have achieved breakthrough results in many tumors, such as melanoma and non‐small cell lung cancer, which caused a paradigm shift of cancer treatment.^3^, ^4^ The US Food and Drug Administration (FDA) has approved PD‐1 inhibitors (e.g., pembrolizumab) for gastrointestinal cancers, including advanced esophageal, gastric, colorectal, hepatic, and pancreatic cancers.^5^ However, the efficacy of current ICB therapies remains a clinical challenge for gastrointestinal cancers owing to the insufficient response to ICB‐based immunotherapy.^6^ To date, ICB therapies are appropriate for gastrointestinal cancers of rare subtypes, such as those having microsatellite instability high (MSI‐H) and DNA mismatch repair deficiency (dMMR), and these molecular subtypes occur in only 5%–10% of all gastrointestinal cancers, with benefits for only a small subset of patients.^7^ Recent clinical trials have revealed that the efficacy of ICB therapies is limited in patients with microsatellite stability, particularly in colorectal and pancreatic cancers.^8^, ^9^, ^10^ Moreover, approximately 50% of cancers with the MSI‐H subtype are intrinsically resistant to anti‐PD‐1 therapies.^11^ Therefore, the small applicability and widespread resistance to ICB therapies drastically limit the efficacy of immunotherapy in gastrointestinal cancers.

Accumulated evidence has revealed the tumor‐intrinsic and tumor‐extrinsic mechanisms of resistance to immunotherapy. Intrinsic factors are characteristic of genetic T‐cell exclusion, absence of antigenic proteins, and antigen presentation, whereas extrinsic factors are immunosuppressive cells, inhibitory immune checkpoints, and absence of T cells,^12^, ^13^ which implies that finding ways to modulate both intrinsic and extrinsic factors is significant for overcoming immunotherapy resistance in gastrointestinal cancers. Metabolic reprogramming, autophagy, and ferroptosis have been demonstrated as critical factors in tumorigenesis and progression of gastrointestinal cancers,^14^, ^15^, ^16^ and are involved in the regulation of tumor trafficking, death, differentiation, activation, and efficacy of immune cell subsets.^17^, ^18^, ^19^ Moreover, metabolic reprogramming can support the unrestrained proliferative and malignant phenotype of cancer cells, while autophagy and ferroptosis can alter the death modalities of tumor and immune cells, enhancing their malignancy. These three pathways play crucial roles in the malignancy of cancer cells, immune cell activity, and the establishment of an immunosuppressive microenvironment.^17^, ^18^, ^19^ Targeting these pathways presents a promising strategy for enhancing the regulation of antitumor immune responses and overcoming resistance to immunotherapy.

There are close interactions between metabolic remodeling, autophagy, and ferroptosis, understanding the crosstalk among these processes is valuable for developing new strategies for gastrointestinal immunotherapy resistance.

In this review, we discuss the effects of metabolic remodeling, autophagy, and ferroptosis on antitumor immune responses in gastrointestinal cancers, the roles of their interactions in immunotherapy, and the potential of these targets in immunotherapy. Improving our understanding of the regulation of these factors and antitumor immunity responses will help develop combination therapies to overcome resistance to gastrointestinal cancer immunotherapy.

## 2 METABOLIC REPROGRAMMING, AUTOPHAGY, AND FERROPTOSIS IN ANTITUMOR IMMUNITY

### 2.1 Metabolic reprogramming in cancer and immune cells

Metabolic reprogramming, a hallmark of cancer, supports unrestricted proliferation, division, and metastasis of tumor cells by rewiring many metabolic pathways, such as glucose, amino acids, and lipids.^20^ Previously excellent reviews have also illustrated that metabolic rewiring promotes tumorigenesis, progression, metastasis, and treatment resistance in gastrointestinal cancers.^21^, ^22^ The metabolic pattern of tumor cells is complex, with obvious heterogeneity, and different metabolic adaptation phenotypes appear owing to changes in the external environment. For example, Metastasis associated in colon cancer 1 (MACC1) is significantly upregulated to facilitate the Warburg effect and ensure gastric cancer growth in glucose deprivation‐induced metabolic stress.^23^ Under nutrient‐deprived conditions, hepatic cancer cells activate the serine biosynthesis pathway to promote cancer progression by upregulating oncogene cMyc.^24^ In addition, this complex and variable metabolic pattern also exists in immune cells, which determines their differentiation and function.^25^ For T‐cell subsets, effector T cells prefer the glycolytic pathway for effect killing function,^26^ whereas other T cells (naive, Treg, memory) usually use fatty acid oxidation (FAO) and oxidative phosphorylation (OXPHOS) pathways to maintain their survival.^27^ Moreover, neutrophils, natural killer (NK) cells, B cells, M1 macrophages, and active dendritic cells (DCs) use the glycolytic pathway as energy supply, whereas M2 macrophages and resting DCs rely on the OXPHOS pathway.^28^, ^29^, ^30^, ^31^ Both tumor and immune cells need to obtain sufficient energy to survive or function through metabolic rewiring, especially glycolysis, implying that competition for nutrients between tumor and immune cells promotes immunosuppressive phenotypes.

### 2.2 Glycolysis

**FIGURE 1:**
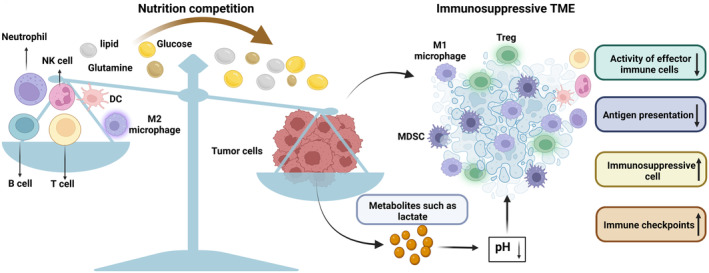
Impact of metabolic reprogramming on antitumor immunity. Tumor metabolic reprogramming, through nutrient competition involving glucose, glutamine, lipids, and other substances, coupled with the export of metabolic byproducts like lactate into the microenvironment, fosters an immunosuppressive tumor microenvironment (TME). This landscape is characterized by diminished effector immune cell activity, reduced antigen presentation, and an increased presence of immunosuppressive cells and immune checkpoints.

Aberrant glucose metabolism is one of the main causes of ineffective antitumor immunity (Figure 1). Chang et al.^32^ revealed that competitive tumor glucose consumption limits the functions of CD8^+^ T effector cells, which leads to the inefficiency of interferon‐gamma (IFNγ) production and failure of tumor clearance. Furthermore, hypoxia‐mediated metabolic rewiring upregulates the glucose metabolism of pancreatic cancer, and glucose consumption inhibits the activity of antitumor T cells.^33^ Additionally, glucose consumption induced by accelerated glycolysis in cancer cells further inhibits effector T‐cell function by inducing Forkhead box protein 3 (FOXP3) expression and promoting infiltration of myeloid‐derived suppressor cells (MDSCs).^34^, ^35^ Thus, targeting gastrointestinal cancer‐specific energy metabolism to suppress the nutrient deprivation of immune cells by tumors is a promising approach to improve the efficacy of immunotherapy.

Glucose transporter proteins (GLUT) are responsible for glucose transport and uptake. As a member of the GLUT family, GLUT‐1 is overexpressed in pancreatic cancer and correlates with a higher density of PD‐1^+^ T cells.^36^ GLUT3 is overexpressed in gastrointestinal cancers^37^ and contributes to the T helper 17 cell responses to M2 macrophage subtype transition.^38^, ^39^ Hexokinase (HK), phosphofructokinase (PFK), and pyruvate kinases (PKs) are rate‐limiting enzymes of the glycolysis pathway and are closely associated with the progression of gastrointestinal cancers.^40^ The overexpression of HK2 is positively correlated with immune infiltration in esophageal cancer; this downregulates the human leukocyte antigen (HLA) of hepatic cancer and impairs the sensitivity of NK cells and innate immune responses.^41^, ^42^, ^43^ The enzyme fructose‐2,6‐bisphosphatase‐3 (PFKFB‐3) plays an essential role in the progression and resistance to sorafenib and upregulates PD‐L1 expression of macrophages to confer immune tolerance in hepatic cancer.^44^, ^45^ Pyruvate kinase isoform M2 (PKM2), the last rate‐limiting enzyme and driver of gastrointestinal cancer, promotes hepatic cancer progression by inducing an immunosuppressive microenvironment,^46^ and the inhibition of PKM2 suppresses PD‐L1 expression on tumor cells.^47^ Moreover, an increase in glycolysis accelerates the production of lactate and decreases OXPHOS. Lactate is then transported into the extracellular environment by monocarboxylate transporter 4 (MCT4),^47^ resulting in an acidic tumor microenvironment (TME) and PD‐1‐overexpressing Treg, which inhibits antitumor immunity and immunosurveillance by T and NK Cells.^48^ In addition, many proteins, long noncoding RNAs (lncRNA), and microRNAs were identified as regulators of glycolysis that modulate antitumor immune response in gastrointestinal cancers. The characteristics of these targets are summarized in Table 1.

**TABLE 1:** Characteristics of molecules that regulate antitumor immune response by modulating metabolism, autophagy and ferroptosis in gastrointestinal cancers.

Metabolic pathways	Cancer type	Phenotype	Target	References
Glycolysis	GC	Activity of γδT cells, macrophages/CD8^+^ T cells exhaustion	H19/CXCL1	49, 50
	CRC	T‐cell proliferation and activation/CD8^+^ T‐cell effector function	ZFP91/NIK	51, 52
		Treg identity and function	MondoA	^53^
	HCC	Improvement of anti‐PD‐1 therapy	CircRHBDD1	^54^
		Upregulation of PD‐L1	miR‐675‐5p	^43^
		M2 macrophage differentiation/MDSCs recruitment	ECT2/PIWIL1	55, 56
	PC	Function of CD4+ T cells	DKK3	^58^
		Immunosuppression in TME	Bmi1‐UPF1‐HK2 axis/BHLHE40	59, 60
Glutamine metabolism	GC	Immune infiltrates in TME	SLC1A5	^61^
	HCC	Immune Infiltration defect	SLC38A1	^62^
		PD‐L1+ exosomes activity	HMGB1/RICTOR	^46^
Lipid metabolism	ESC	Macrophage alternative activation	PPARγ	^63^
	CRC	Regulation of IL‐10‐producing regulatory B cells	LXA4	^64^
		Regulation of IFN‐γ	FABP1	^65^
		Regulation of PD‐L1	USP19	^66^
	HCC	Regulation of IFN‐γ	SREBF1/miR‐27a	57, 67
		Regulation of IFNGR1 and MHC‐I expression	Optineurin	^68^
		MDSCs recruitment	CCRK	^69^
		Regulation of Treg cells/HLA‐C and B2M expression	MARC2	^70^
		CD8+ T‐cell proliferation/T‐cell infiltration and activation	CD137/ARF1	71, 72
	PC	T‐cell survival and persistence	ACADVL	^73^
Autophagy	GC	Tregs infiltration	BECLIN‐1	^74^
	CRC	Function of DCs	HMGB1	^75^
		Tregs infiltration	SQSTM1	^76^
		Regulation of IFN‐I	ATG16L1	^76^
	HCC	CD8+ T‐cell suppression	GOLM1	^77^
		Induction of acidic/immunostimulatory TME	ASIC1a/LIF	78, 79
	PC	MHC‐I expression	NBR1	80, 81
		CD8+ T‐cell infiltration and effector function	RNF31	^82^
		PD‐L1 expression/M2 macrophage differentiation	SEMA3C	^83^
Ferroptosis	ESC	CD8+ T Cells apoptosis	LncRNA‐OIP5‐AS1	^84^
	GC	Regulation of T/B cell reporter/immune checkpoints	SLC2A3	^85^
	CRC	Immune infiltration in TME	SLC2A1/MT1G	86, 87
		Leukocytes recruitment, T‐cell accumulation	OTUD1	^88^
	HCC	Immune infiltration in TME	BTBD10/HSPB1	89, 90
	PC	Immune infiltration in TME, tumor mutation burden	Linc02432, Hsa‐miR‐98‐5p, HK2	^91^

Abbreviations: CRC, colorectal cancer; ESC, esophageal cancer; GC, gastric cancer; HCC, hepatocellular carcinoma; PC, pancreatic cancer.

### 2.3 Glutamine metabolism

Another striking feature of cancer metabolic reprogramming is glutamine addiction. Glutamine is essential for redox balance, de novo synthesis of nucleotides, efficient DNA replication, and cell proliferation in cancer,^61^ and increased glutamine consumption can exhaust nutrients within the tumor, leading to metabolic stress that may affect the progression of gastrointestinal cancer.^92^, ^93^ In addition, immune cells require glutamine uptake for survival, activation, and differentiation. Glutamine can promote the transcription of proliferation‐related genes by activating the ERK and JNK signaling pathways, resulting in the rapid proliferation of immune cells.^94^ Glutamine metabolism is also essential to regulate the activation of macrophages and facilitate the differentiation of M2 macrophages.^94^ In TME, tumor and immune cells also compete for glutamine uptake, similar to the competition between glucose. Selective inhibition of glutamine metabolism in tumor cells can increase glutamine uptake of T cells and enhance their antitumor activity,^95^ and the activation of the MAPK/ERK pathway modulates glutamine uptake between tumor and T cells.^96^ A recent study reveals that glutamine allocation in TME is programmed, with cell‐intrinsic programs driving tumor cells to preferentially consume glutamine, whereas immune cells consume glucose.^97^ This nutrient distribution strategy is induced by mTORC1 signaling‐mediated expression of glucose‐ and glutamine‐related genes. Thus, these cell‐intrinsic programs may be potential clinical translation targets to overcome immunotherapy resistance in gastrointestinal cancers.

In addition to glutamine status in the TME, glutamine metabolic reprogramming in tumor cells also significantly affects antitumor immune responses. Glutamine deprivation in tumor cells activates EGFR/ERK/C‐JUN signaling and then upregulates the expression of PD‐L1 in tumor cells, which weakens the antitumor immune response.^98^ Glutamine metabolic reprogramming promotes tumorigenesis of hepatic cancer, upregulates the expression of lymphocyte activation gene 3 protein (LAG3) in infiltrating γδ T cells,^99^ and impairs immunotherapy by PD‐L1.^98^ Moreover, in a mouse model of colon cancer, glutamine blockade decreases hypoxia, acidosis, and nutrient depletion, and destroys immunosuppressive TME to overcome tumor immune evasion.^99^ Sharma et al.^100^ have shown that targeting glutamine‐utilizing enzymes using a glutamine analog (6‐diazo‐5‐oxo‐l‐norleucine) can remodel the extracellular matrix (ECM) and elevate infiltration of CD8^+^ T cells, leading to sensitization of pancreatic cancer to anti‐PD1 therapy. Glutamine transporter SLC1A5 is also positively associated with immune infiltrates in gastric cancer,^101^ and inhibition of SLC1A5 improves antitumor immunity in TME.^102^ Therefore, targeting cancer‐specific glutamine metabolism is a promising strategy to improve the efficacy of immunotherapy. Other molecules involved in the antitumor immune response by modulating glutamine metabolic reprogramming in gastrointestinal cancers are shown in Table 1.

### 2.4 Lipid metabolism

Tumor cells usually exhibit a high affinity for fatty acids and cholesterol to support their growth, and many lipogenic enzymes, such as fatty acid synthase (FASN), ATP‐citrate lyase (ACLY), and acetyl‐CoA carboxylase (ACC), are overexpressed in gastrointestinal cancers.^103^ In contrast, fatty acids and lipid accumulation result in general immunosuppression in immune cells. For example, high amounts of lipids stimulate M2 macrophages, leading to protumorigenic polarization in gastric cancer,^104^ long‐chain fatty acid metabolism reinforces the suppression by tumor‐associated macrophages of tumor immune surveillance.^105^ Moreover, lipid accumulation in DCs inhibits its capacity to process antigens.^106^ Fatty acids are essential for the differentiation of Treg cells, and the overexpression of lipid transporter CD36 contributes to the survival and functions of Treg cells.^107^ The accumulation of long‐chain fatty acids also damages CD8^+^ T‐cell functions in pancreatic cancer.^73^ Moreover, pharmacological inhibition of FAS using an ACC inhibitor reduces lipid levels in DCs and rescues their antitumor immunity activity,^106^ and inhibition of FASN protects CD4^+^ effector T cells from restimulation‐induced cell death and enhances T‐cell immunity.^108^ FAO blockade also limits the immunosuppressive effect of M2 macrophages and Tregs.^109^, ^110^

The metabolism of cholesterol represents a pivotal pathway in modulating antitumor immunity. High levels of cholesterol induce overexpression of PD‐1, LAG‐3, and TIM3 in immune cells, which perturbs its normal lipid metabolism, promotes its apoptosis, and reduces its proliferation.^111^, ^112^ Free cholesterol in the T‐cell membrane is an integral component of the T‐cell receptor (TCR) and the immunological synapse of T cells, directly orchestrating signaling pathways and effector functions.^113^ Gu et al.^114^ found that cholesterol esterase acetyl‐CoA acetyltransferase 1 (ACAT1), an oncogene, promotes hepatocarcinogenesis by remodeling lipid metabolism, and pharmacological inhibition using avasimibe could have an antitumor effect on CD8^+^ T cells and improve the efficacy of PD‐1 inhibitors by regulating cholesterol metabolism.^115^ Within the tumor microenvironment, lactate suppresses the expression of PPARγ in intratumoral iNKT cells, thereby diminishing their cholesterol synthesis and production of IFN‐γ, attenuating the antitumor efficacy of iNKT cells in hepatocellular carcinoma.^57^ Conversely, ATP‐binding cassette transporter A9 promotes cholesterol accumulation in macrophages, amplifying antitumor immunity against peritoneal metastasis in colorectal cancer.^116^ Elevated serum cholesterol levels enhance the antitumor capabilities of natural killer cells and decelerate the growth of murine liver tumors.^117^ Absence of Apolipoprotein E (ApoE) results in a reduced proportion of MDSCs in the body, leading to accelerated therapeutic growth.^118^ Notably, both an increase or decrease in cholesterol can inhibit T‐cell function.^113^ These findings underscore a dual role of cholesterol metabolism in resistance to immunotherapy for gastrointestinal tumors, suggesting that targeting cholesterol metabolic imbalances might be a potential avenue to rejuvenate antitumor immune efficacy.

Moreover, lipid metabolism‐related genes are also closely associated with immune infiltration. The modulation of lipid metabolism through ADP ribosylation factor 1 (ARF1) curtails the release of damage‐associated molecular patterns (DAMPs), which compromise antitumor immune surveillance by suppressing T‐cell infiltration and activation in CRC.^119^ Gu et al.^114^ and Hu et al.^120^ have established lipid metabolism‐based prediction models that characterize the relationship between lipid metabolism‐related genes, immune infiltration, and prognosis in colon cancer and hepatocellular carcinoma. We generalized the mechanisms by which lipid metabolism regulates antitumor immunity and summarized the molecules that enhance or weaken antitumor immunity by regulating lipid metabolism (Table 1). Together, this complex fatty acid and cholesterol metabolism offers an opportunity to modulate the immunosuppressive TME and enhance antitumor immunity.

### 2.5 Autophagy

**FIGURE 2:**
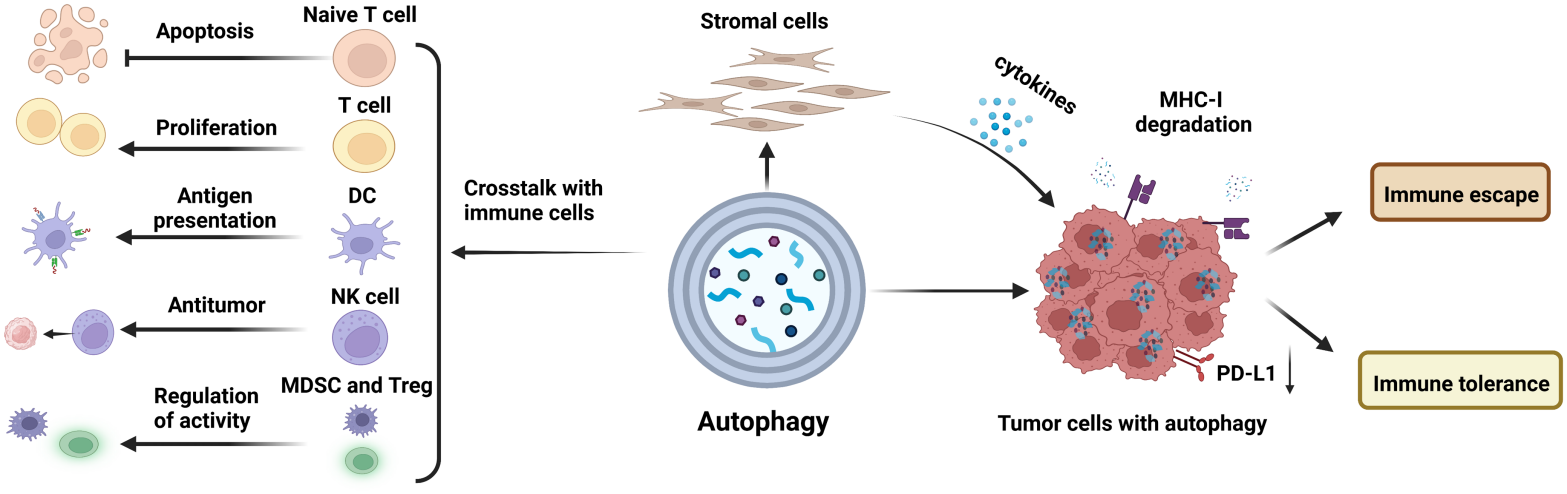
Impact of autophagy on antitumor immunity. Autophagy modulates the functions of naive T cells, T cells, dendritic cells (DC), natural killer (NK) cells, myeloid‐derived suppressor cells (MDSC), and regulatory T cells (Treg). Additionally, it promotes the secretion of cytokines by stromal cells and facilitates MHC‐I degradation and PD‐L1 downregulation in tumor cells, culminating in immune evasion and immune tolerance.

Autophagy is a highly conserved multistep catabolic pathway dominated by macroautophagy (hereafter referred to as autophagy) to maintain metabolic adaptation, which is dependent on intracellular lysosomes and is regulated by the ATG family of proteins. Autophagy, as a “double‐edged sword,” plays a complex role in gastrointestinal tumor biology. For instance, autophagy as a suppressor delays cancer cell transformation during pancreatic and liver tumorigenesis, whereas in transformed cancer cells, it promotes the progression, treatment, and treatment resistance of hepatic and pancreatic cancer.^121^, ^122^, ^123^ In addition, autophagy regulates the activity of immune cells and stromal cells in the TME and exacerbates immune evasion and tolerance of tumor cells (Figure 2). Therefore, autophagy plays an important role in regulating resistance to immunotherapy.

### 2.6 Autophagy in the TME

Autophagy can promote or inhibit tumor progression by regulating the survival, activation, proliferation, and differentiation of immune cells in the TME. Xia et al.^124^ have reported that autophagy deficiency induces naive T‐cell apoptosis and inhibits tumor immunity, and autophagy‐related protein phosphatidylinositol 3‐kinase catalytic subunit type 3 (PIK3C3) deficiency impairs T‐cell mitochondrial metabolism, resulting in the failure of CD4^+^ T cells to differentiate into effector T cells.^125^ Moreover, defective proliferation is observed in ATG3/ATG5/PIK3C3‐deficient T cells by facilitating the accumulation of cell‐cycle inhibitor cyclin‐dependent kinase inhibitor 1B (CDKN1B),^126^ and Autophagy‐related 5/7 (ATG5/ATG7) deficiency causes functional deficit of memory CD8^+^ T cells.^127^ Autophagy also plays a critical role in the antigen processing and presentation functions of DCs. ATG5 deficiency in DCs upregulates the scavenger receptor CD36 and positively regulates major histocompatibility complex class II (MHC‐II) antigen presentation,^128^ and autophagy‐related protein Vps34 is essential for functions of antigen cross‐presenting CD8α^+^ DCs.^129^ As a major innate effector component, NK cells exert powerful antitumor immunity. Phosphorylated Forkhead box O1 (FOXO1) interacts with ATG7 and induces autophagy in immature NK cells, and this FOXO1‐mediated autophagy is essential for NC cell‐induced antitumor immunity.^130^ As professional antigen‐presenting cells, B cells require autophagy to regulate antigen presentation, cross‐presentation, and memory maintenance functions.^131^ The inhibition of autophagy promotes the polarization of hepatoma‐related M2 macrophages and elevates antitumor phagocytosis and cytotoxicity of macrophages.^132^ Moreover, autophagy is essential to regulate the survival and function of immunosuppressive Tregs and MDSCs. For example, the inhibition of autophagy promotes the activation of STAT3 signaling, resulting in the accumulation and immunosuppressive function of MDSCs.^133^ Autophagy also enhances the functional integrity of Tregs by maintaining homeostasis between environmental stress and metabolic adaptations.^134^ In addition, stromal cells in the TME secrete specific cytokines through autophagy to promote autophagy of tumor cells and accelerate tumor progression. Li et al.^135^ revealed that hypoxia‐induced autophagy of stellate cells reduces stromal lumican in the TME of pancreatic cancer, thereby promoting cancer progression. Wang et al.^136^ also found that cancer‐associated fibroblasts promote the survival of irradiated cancer cells by secretory factor‐induced autophagy. Furthermore, an intricate dance exists between DAMPs and autophagy. By thwarting autophagy utilizing hydroxychloroquine, the vigor of DAMPs is heightened, galvanizing dendritic cell activation. In a counter‐play, DAMPs might spur autophagy, thus advancing tumor malignancy.^137^ Therefore, targeting autophagy in the TME may be a novel approach to circumvent TME‐mediated immunotherapy resistance.

### 2.7 Autophagy in tumor cells

In addition to autophagy in the TME, autophagy in tumor cells can also affect antitumor immunity. Altered expression of MHC‐I in tumor cells usually helps them evade immune surveillance, which causes immunotherapy resistance. Among gastrointestinal tumors, pancreatic cancer is the least sensitive to immunotherapy. Recent studies have illustrated that autophagy facilitates immune evasion of pancreatic cancer by lysosomal‐mediated degradation of MHC‐I, and inhibition of autophagy using chloroquine rescues the expression of MHC‐I, improves antigen presentation, and synergizes with immunotherapy (anti‐PD1 or anti‐CTLA4 antibodies).^80^, ^81^ Pharmacological inhibition of MEK and autophagy activate STING/TNF‐1 signaling and paracrine signaling, in turn promoting tumor‐associated macrophages toward an immunogenic M1‐like phenotype, leading to the activation of immune recognition in pancreatic cancer.^138^ Moreover, during chemotherapy‐induced immunogenic cell death in colon cancer cells, ATG5/ATG7 deficiency impairs antitumor immunity by reducing the release of adenosine triphosphate.^139^ Autophagy has also been shown to regulate the expression of immune checkpoints in tumor cells to promote immune tolerance. For example, the inhibition of autophagy upregulates the expression of PD‐L1 in gastric cancer cells and enhances the efficacy of PD‐L1 blockade.^140^ Thus, autophagy plays critical roles in the regulation of the antitumor immune response by modulating MHC‐I‐antigen complexes, immunogenic tumor cell death, and expression of immune checkpoints in gastrointestinal cancers. In addition, autophagy has a twofold function in both the initial emergence and advanced stages of cancer. In the early stages, autophagy suppresses the process of carcinogenesis by degrading toxic substances within cells and by regulating intercellular communication mediated by proteins and hormones. However, in advanced stages, autophagy promotes cancer progression by recycling cellular components to provide metabolic substrates, facilitating the metabolic reprogramming of cancer cells.^141^ We summarize the characteristics of molecules that regulate autophagy and antitumor immunity in Table 1.

### 2.8 Ferroptosis

**FIGURE 3:**
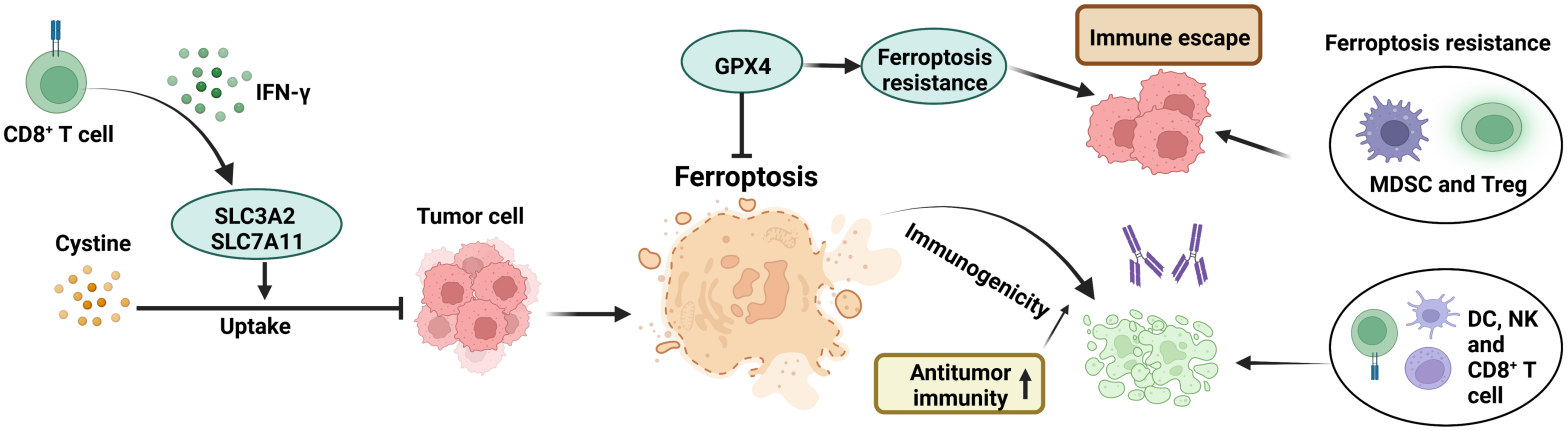
Impact of ferroptosis on antitumor immunity. Activated CD8^+^ T cells can downregulate the expression of SLC3A2 and SLC7A11 via the release of IFN‐γ, inhibiting the uptake of cystine by tumor cells. This promotes ferroptosis in tumor cells, enhancing their immunogenicity. CPX4 fosters immune evasion by attenuating the sensitivity to ferroptosis.

Ferroptosis is a form of programmed cell death characterized by lethal levels of iron‐metabolism‐dependent accumulation of lipid reactive oxygen species (ROS). Recently, numerous studies have shown that ferroptosis is closely related to the antitumor immune regulation of gastrointestinal cancers. A predictive model of immune response in hepatocellular, pancreatic, colon, and gastric cancers has been constructed based on ferroptosis‐related LncRNAs.^142^, ^143^, ^144^, ^145^ Ferroptosis act as a key regulator of survival and activation of immune cells in the TME. The selenoenzyme glutathione peroxidase (GPX4) is a central repressor of ferroptosis, and overexpression of GPX4 renders CD8+ T cells ferroptosis‐resistant and protects against death induced by excess lipid accumulation. The inhibition of ferroptosis in CD8^+^ T cells effectively restores their antitumor activity.^146^, ^147^ Hou et al.^84^ reported that GPX4 reduces the proportion of CD8^+^ T cells and promotes immune evasion of esophageal cancer cells by alleviating ferroptosis. Moreover, immunosuppressive MDSCs and Tregs both exhibit high ferroptosis resistance that reduces antitumor immunity.^148^ Wang et al.^149^ first explicitly reported the phenomenon of crosstalk between the ferroptosis pathway of immune cells and tumor cells; immune‐activated CD8^+^ T cells downregulate the expression of glutamate‐cystine anti‐transport system proteins (SLC3A2 and SLC7A11) by releasing IFN‐γ, inhibiting the uptake of cystine by tumor cells, and promoting tumor cell lipid peroxidation and ferroptosis, resulting in enhanced antitumor immunity. The induction of ferroptosis in tumor cells also increases their immunogenicity and improves the efficacy of anti‐PD‐1 therapy.^150^ Zhang et al.^151^ found that dihydroartemisinin induces ferroptosis in pancreatic cancer and contributes to an increase in the population of CD8^+^T, NK, and NKT cells. Additionally, early cellular ferroptosis induces immunogenic cell death, and concurrently, the release of DAMPs leads to the maturation of DC.^137^ Therefore, the induction of ferroptosis enhances the killing effect of immune cells on tumor cells and inhibits the survival of immunosuppressive cells while reducing the antitumor activity of effector T cells (Figure 3). However, tumor cells are more sensitive to ferroptosis than effector T cells,^148^ suggesting that this approach is a promising antitumor strategy. The features of molecules that regulate ferroptosis and modulate antitumor immunity are shown in Table 1.

## 3 CROSSTALK BETWEEN METABOLIC REPROGRAMMING, AUTOPHAGY, AND FERROPTOSIS

Metabolic reprogramming, autophagy, and ferroptosis play critical roles in the regulation of cancer progression and antitumor immunity, and crosstalk exists between these three processes in antitumor immunity. Autophagy recycles intracellular components under metabolic stress and regulates the energy metabolism of immune cells in the TME. Recent results suggest that induction of ferroptosis is dependent on autophagy and metabolic reprogramming.^152^, ^153^ Therefore, an in‐depth understanding of the crosstalk among the three is conducive to the discovery of new antitumor immunotherapy strategies.

### 3.1 Autophagy and metabolic reprogramming

The main role of autophagy is to provide sufficient substrate for anabolism. Autophagy‐deficient tumor cells are incapable of macromolecular degradation and Krebs cycle substrate support, which prevents aspartate production and nucleotide synthesis.^141^ Autophagy also promotes mitochondrial metabolism and removes mitochondria with excessive levels of oxidative stress, and this crosstalk is convenient for tumor growth.^154^ Autophagy is activated in the presence of hypoxia and nutrient deprivation in the TME.^155^ Under hypoxia, tumor cells evade immune surveillance by T cells by inducing autophagy through HIF‐1α/STAT3 signaling,^156^ and hypoxia‐induced autophagy reduces NK cell killing by degrading the NK‐derived serine protease granzyme B (GZMB) during transport within cancer cells.^157^ Moreover, autophagy of cancer‐associated fibroblasts (CAFs) directly supplies lactate, ketone bodies, fatty acids, and glutamine for cancer cell proliferation in the TME.^158^ In addition, the phosphatase and tensin homolog deleted on chromosome 10 (PTEN), a tumor suppressor, suppresses glycolysis, the pentose phosphate pathway, lipid synthesis, and pyrimidine synthesis by blocking AKT and mTOR signaling.^159^ PTEN also induces autophagy by inhibiting mTOR signaling from regulating epithelial to mesenchymal transition (EMT) and invasion of cancer cells.^159^ As a common switch for autophagy and metabolic reprogramming, PTEN achieves tumor suppression and immune protection by blocking mTOR signaling, inducing T‐cell infiltration, inhibiting M2 macrophage polarization, and decreasing PD‐L1 expression in the TME,^159^, ^160^, ^161^ suggesting that PTEN is a promising therapeutic target to enhance antitumor immunity.

### 3.2 Metabolic reprogramming and ferroptosis

A recent study has demonstrated a dynamic interaction between ferroptosis and glycolysis, lipid metabolism, and amino acid metabolism in the TME, which affects the efficacy of immunotherapy.^162^ Increased glycolysis in tumor cells and CAFs produces excess lactate, which creates an acidic TME, leading to immune escape, tumor metastasis, and therapy resistance. Pucino et al.^163^ reported that lactate accumulation regulates ferroptosis by modulating the production of lipids. Inhibition of hydroxycarboxylic acid receptor 1 (HCAR1) or monocarboxylate transporter 1 (MCT1) blocks lactate uptake and reduces ATP production in hepatocellular carcinoma cells, resulting in the activation of AMPK signaling and ferroptosis.^164^ This suggests that this crosstalk between lactate and ferroptosis reshapes the TME and may lead to immunosuppressive and immune evasion phenotypes. Lipid metabolism is closely related to the sensitivity of cells to ferroptosis and plays an important role in antitumor immune regulation. Cancer cells in ferroptosis regulate the activity of immune cells in the TME by releasing oxidized lipid metabolites such as prostaglandin E2 (PGE2).^165^ As an immunosuppressive factor, PGE2 inhibits the antitumor effect of NK cells, DCs, and CD8^+^ T cells in the TME.^166^ In addition, amino acid metabolism is an important step in regulating ferroptosis; it enhances the antioxidant capacity of cancer cells by providing cysteine, cystine, and glycine to combat ferroptosis. Cystine deficiency impairs mitochondrial OXPHOS by inhibiting cytosolic aspartate aminotransaminase (GOT1), inducing ferroptosis‐mediated cell death in pancreatic cancer.^167^ The cystine‐glutamate antiporter xCT protein SLC7a11 regulates cellular sensitivity to ferroptosis by regulating glutathione synthesis, and inhibition of SLC7a11 improves the efficacy of anti‐CTLA‐4 in the colon and pancreatic cancers.^168^

### 3.3 Autophagy and ferroptosis

Autophagy is an important pathway for cells to maintain homeostasis, but excessive or no autophagy induces the appearance of the “autophagy‐dependent death” phenotype. Ferroptosis is an iron‐dependent form of lipid peroxidation‐mediated cell death that requires autophagy‐mediated regulatory mechanisms.^169^ Remarkably, several key regulators of ferroptosis, including SLC7A11, GPX4, NRF2, and p53, also hold crucial roles in the orchestration of autophagic processes. For instance, GPX4 has been delineated to directly engage with copper, consequentially inducing the aggregation of GPX4 and its subsequent degradation via autophagic pathways—a pivotal process steering ferroptosis.^170^ Chaperone‐mediated autophagy can further catalyze the degradation of GPX4, serving as an ignition for ferroptosis.^171^ This crosstalk between autophagy and ferroptosis is closely associated with cancer progression and antitumor immune response. Cells in ferroptosis release proteoglycan decorin (DCN) dependent on autophagy, leading to pro‐inflammatory pathology, and the inhibition of DCN release limits the ability of pancreatic cancer cells in ferroptosis to induce a cancer‐protective immune response.^172^ Chen et al.^173^ revealed that the interplay between autophagy and ferroptosis is vital for the regulation of TME immunity, treatment resistance, and prognosis. Liu et al.^174^ also identified transmembrane protein 164 (TMEM164) as a key regulator of autophagy‐dependent ferroptosis, and the overexpression of TMEM164 is closely associated with the improvement of survival outcome and immune infiltration in patients with pancreatic cancer. In addition, pancreatic cancer cells with autophagy‐dependent ferroptosis release the KRAS^G12D^ protein, which is taken up by macrophages, resulting in the polarization of M2 macrophages, which promotes the progression of pancreatic cancer.^175^

**FIGURE 4:**
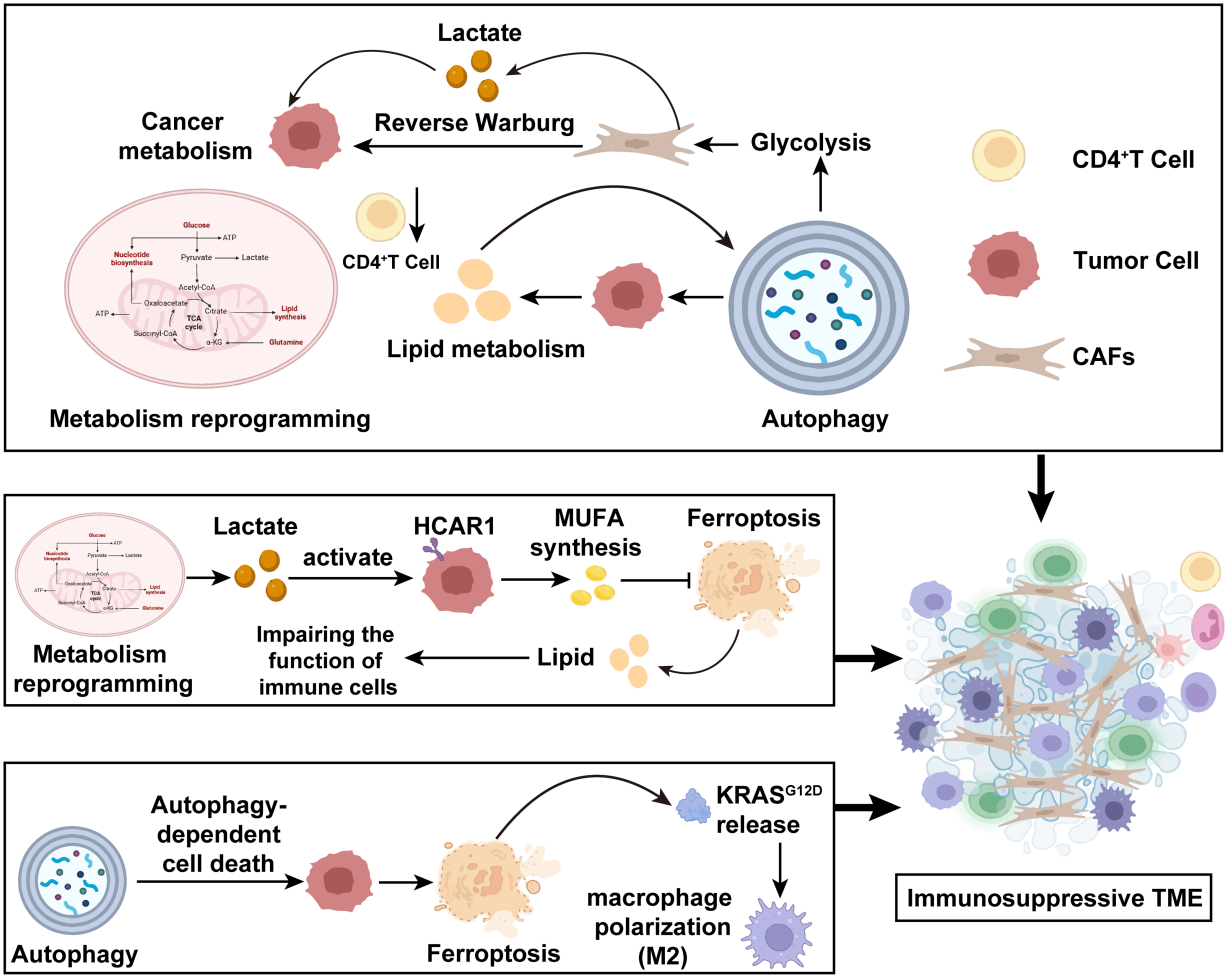
Interplay of metabolic reprogramming, autophagy, and ferroptosis in promoting an immunosuppressive TME. Autophagy instigates the glycolytic pathway in CAFs, mediating the reverse Warburg effect, and further establishes a feedback loop with lipid metabolism. Metabolic reprogramming, through lactic acid release, activates HCAR1, promoting MUFA synthesis, leading to ferroptosis. The lipids released subsequently dampen the activity of immune cells. Autophagy‐mediated cell death further accelerates ferroptosis in tumor cells, resulting in the release of KRAS^G12D^ protein and activation of M2 macrophages. These cascading events collectively sculpt an immunosuppressive TME.

The intricate dance of autophagy, metabolic reprogramming, and ferroptosis intricately weaves a tapestry that establishes an immunosuppressive TME. Within this complex nexus, metabolic perturbations in the TME actuate the autophagic machinery in CAFs, nudging them toward a glycolytic metabolic predisposition.^176^ This metabolic pivot, evoking echoes of a reverse Warburg effect, orchestrates a concerted extrusion of lactate into the TME, providing tumor cells with an essential metabolic lifeline.^176^ Particularly in hepatocellular carcinoma, an intrinsic autophagic drive fosters lipid droplet accretion and curtails the presence of CD4^+^ T cells, embedding the immunosuppressive tenor of the TME even further.^177^ Furthermore, a heightened orchestration of lipid metabolism resonates synergistically with autophagy, collaboratively modulating the energetic harmonics of the TME.^178^ Central to this, the nuanced choreography between autophagy and metabolic reprogramming potentially creates a reverberating feedback loop, amplifying the immunosuppressive ambiance of the TME, paving the way for immunotherapeutic recalcitrance. As tumors evolve, cells conspicuously adopt a heightened glycolytic metabolic signature, catalyzing a prolific release of lactic acid that subsequently sculpts the TME's architecture. This amassed lactic acid serves a pivotal role as a molecular activator, zeroing in on and invigorating the hydroxycarboxylic acid receptor 1 (HCAR1) ensconced on the tumor cell surface. The ensuing activation of HCAR1 champions the synthesis of monounsaturated fatty acids (MUFA), staunch defenders against ferroptosis. It is imperative to underscore that ferroptotic cells exude lipid metabolites that possess the capability to hamstring the functionality of immune sentinel cells, thereby nurturing the seeds of an immunosuppressive microcosm.^179^ In parallel, autophagy‐fueled ferroptosis shepherds the alignment of tumor‐associated macrophages into the M2 paradigm via release and uptake of oncogenic KRAS protein, potentially ushering in adaptive immune subjugation.^175^ In summation, the orchestrated symphony among these tripartite pathways crescendos into an immunosuppressive microenvironmental anthem, unveiling novel prospects for strategizing against immunotherapy resistance (Figure 4).

## 4 TARGETING METABOLIC REPROGRAMMING, AUTOPHAGY, AND FERROPTOSIS

We summarize the antitumor immunity effect of inhibitors targeting the metabolic reprogramming/autophagy/ferroptosis processes in gastrointestinal cancers in clinical development in Table 2. AZD3965, a selective inhibitor targeting MCT1, significantly reduces colon cancer growth by increasing immune infiltration of DC and NK cells.^180^ Sasaki et al.^181^ reported that deoxy‐D‐glucose (2‐DG) induces antitumor immunity by regulating glucose metabolism in the TME of hepatocellular carcinoma. A glutamine analog (6‐diazo‐5‐oxo‐l‐norleucine, DON) and its prodrug DRP‐104 inhibit glutamine anabolism and improve the efficacy of immunotherapy in gastrointestinal cancers.^109^, ^182^ In a patient‐derived xenograft (PDX) model of colorectal cancer, simvastatin reduces cholesterol biosynthesis and elevates antitumor immunity by downregulating PD‐L1 expression.^183^ Pharmacological inhibition of fatty acid transporter protein 2 (FATP2) using lipofermata abrogates the activity of polymorphonuclear MDSC and substantially inhibits tumor progression in a mouse model of pancreatic and colorectal cancers.^184^ Chloroquine (CQ) and hydroxychloroquine (HCQ), autophagy inhibitors, inhibit lysosome acidification and have been demonstrated as regulators of antitumor immunity in pancreatic and colorectal cancers.^80^, ^81^, ^185^ Two phase II clinical studies (NCT04214418 and NCT03344172) also use HCQ as sensitizer of anti‐PD‐1 in gastrointestinal cancer. Erastin is an inhibitor targeting system Xc, a ferroptosis regulator consisting of SLC3A2 and SLC7A11, that induces powerful antitumor immunity by promoting differentiation of Th17 and activating the efficacy of CD8^+^ T cells.^186^, ^187^ Moreover, a recent study also found that sorafenib induces ferroptosis of hepatoma carcinoma cells and enhances antitumor immunity. Therefore, these studies indicate that targeting metabolic reprogramming, autophagy, and ferroptosis are promising strategies for improving the efficacy of immunotherapy in gastrointestinal cancer.

**TABLE 2:** Metabolism/autophagy/ferroptosis inhibitors in clinical development combined with immunotherapy.

Metabolic pathways	Inhibitor	Target	Immune effect	Cancer types	Status/Model	References
Glycolysis	AZD3965	MCT1	Increase of tumor immune infiltration	CRC	Preclinical/CDX	^180^
	2‐DG	GLUT1	Increase of CD8+ T‐cell chemotaxis	HCC	Preclinical/CDX and syngeneic mouse	^181^
Glutamine metabolism	DON	GFAT1	Sensitization for anti‐PD1 therapy	PC	Preclinical/syngeneic mouse	^100^
	DPR‐104	GFAT1	Improvement of immune cell infiltration and effect	CRC and HCC	Preclinical/CDX	^182^
Lipid metabolism	Simvastatin	HMGCR	Downregulation of PD‐L1 expression	CRC	Preclinical/PDX	^183^
	Lipofermata	FATP2	Inactivation of MDSCs	PC, CRC	Preclinical/CDX and syngeneic mouse	^184^
Autophagy	HCQ	Lysosome	Combination with atezolizumab	Gastrointestinal cancer	Clinical trial/Phase II	^NCT04214418^
	HCQ	Lysosome	Combination with avelumab	PC	Clinical trial/Phase II	^NCT03344172^
	CQ	Lysosome	Degradation of MHC‐I, Sensitization for anti‐PD1/CTLA‐4 therapy	PC	Preclinical/CDX and syngeneic mouse	80, 81
	CQ	Lysosome	Induction of DC maturation and T‐cell responses	CRC	CDX	^185^
Ferroptosis	Erastin	System Xc	Differentiation of Th17 cell	HCC	CDX	^186^
	Erastin	System Xc	Activation of CD8^+^ T cells	HCC and CRC	CDX	^187^
	Sorafenib	System Xc	Infiltration of CD8^+^ T cells	HCC	CDX	^188^

Abbreviations: CDX, cell‐derived xenografts; CRC, colorectal cancer; HCC, hepatocellular carcinoma; PC, pancreatic cancer; PDX, patient‐derived xenografts.

## 5 CONCLUSIONS AND PERSPECTIVES

Weak immune response and widespread resistance severely limit the application of immunotherapy in gastrointestinal cancer. Metabolic reprogramming, autophagy, and ferroptosis have recently been identified as key regulators of antitumor immunity. This review delineates the synergistic roles of metabolic reprogramming, autophagy, and ferroptosis in shaping antitumor immunity in gastrointestinal malignancies and unravels the intricate interplay between these processes. While metabolic reprogramming can diminish antitumor immune responses by reshaping the behavior of tumor cells, stromal cells, and immune cells within the tumor microenvironment, its systemic nature necessitates the identification of more specific metabolic patterns in gastrointestinal tumors to counteract immunotherapeutic resistance. Both autophagy and ferroptosis profoundly influence the survival capacities of tumor cells and the activity of immune cells in the microenvironment, but they can antagonize while concurrently synergizing antitumor immune responses. The crosstalk between these pathways plays a critical role in advancing an immune‐suppressive tumor milieu. However, the mechanisms by which this interplay fosters resistance to immunotherapeutic strategies in gastrointestinal cancers remain elusive. Deciphering the nuances of tumor immune evasion and tolerance is pivotal for devising combined therapeutic approaches.

From a clinical vantage point, a diverse array of immunotherapeutic strategies, complemented by drug regimens targeting metabolic reprogramming, autophagy, and ferroptosis, have undergone rigorous evaluation in both preclinical models and advanced clinical trials. These endeavors have manifested in early‐phase results that are not only promising but also indicative of the therapeutic potential these avenues harbor. However, navigating the intricate lattice of these molecular pathways presents inherent challenges. To truly leverage their capabilities and overcome the formidable barriers of immunotherapeutic resistance inherent to gastrointestinal tumors, there is an imperative to delve deeper into the discovery of highly specific regulatory nodes and checkpoints. By doing so, we can mitigate the unintended consequences arising from inadvertently disrupting immune cell dynamics within the tumor microenvironment. Such an approach also promises to illuminate the molecular landscapes that are optimally attuned to these therapeutic strategies, enhancing not only the specificity but also the overall efficacy of these targeted interventions.

## AUTHOR CONTRIBUTIONS

**Xiangwen Wang:** Conceptualization (equal); data curation (lead); formal analysis (equal); investigation (lead); software (lead); validation (lead); writing – original draft (lead). **Liwen Zhou:** Data curation (lead); validation (supporting). **Hongpeng Wang:** Data curation (supporting); software (lead). **Wei Chen:** Data curation (lead); validation (equal). **Lei Jiang:** Data curation (lead); investigation (equal). **Guangtao Ming:** Data curation (equal). **Jun Wang:** Conceptualization (lead); data curation (lead); formal analysis (lead); project administration (lead); writing – review and editing (lead).

## CONFLICT OF INTEREST STATEMENT

The authors declare that they have no conflict of interest.

## ETHICS STATEMENT

Ethics approval statement is not applicable to this article.

## Data Availability

Data sharing is not applicable to this article as no datasets were generated or analyzed during the current study.
